# The promise of eHealth for primary care: opportunities for service delivery, patient–doctor communication, self-management, shared decision making and research

**DOI:** 10.1080/13814788.2018.1449779

**Published:** 2018-03-26

**Authors:** Jelle Stoffers

**Affiliations:** Department of Family Medicine, The European Journal of General Practice, Care and Public Health Research Institute (CAPHRI), Maastricht University, Maastricht, The Netherlands


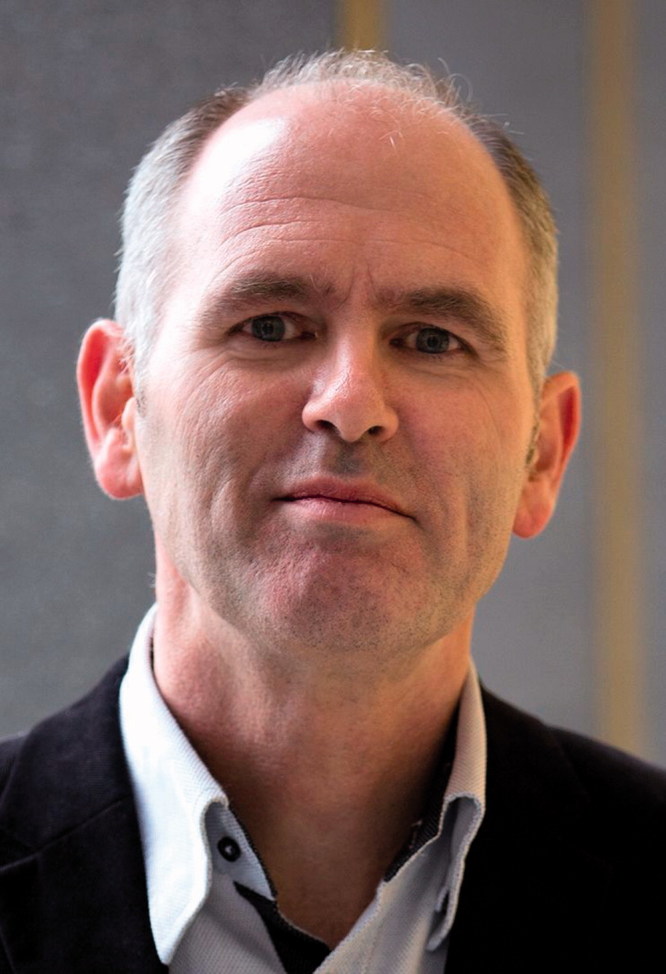
Recently, the ‘eHealth week’ was organised in the Netherlands for the second time. It was a government campaign aimed at citizens (patients) with the objective of increasing the ‘knowledge and skills in the area of digital support in healthcare’. Patients were the focus of this awareness-raising campaign, with activities across the country. For me, it was the start of a fascinating internet inquiry.

I wonder whether general practice/family medicine is not lagging behind? In any case, we do not seem to be front-runners in this area. In the Netherlands, there is a specific funding programme for the eight university medical centres, with one part focused on eHealth. That sub-programme includes research topics like hospital patient portals (with features like medication and personal medical records integrated with the hospital medical record), a specialist medical dashboard for GPs, wearables for home tele-monitoring (home-hospital) integrated with the (hospital) electronic medical record, an app for shared decision making for lung patients, an app for patients and their caregivers on cognitive problems, a dashboard and apps to prepare patients for their talk with their specialist on an important decision (orthopaedic patients, lung patients, cancer patients), a physiotherapy app for patients to do exercises at home, etc. In short, it mainly is about patients measuring, monitoring or preparing themselves at home, communicating through the internet with healthcare professionals (i.e. specialists), and having their data integrated into an electronic medical (hospital) information system. It should support shared decision-making, self-management and efficient healthcare [1].

What about primary care then? The Dutch College of General Practitioners published its ‘point-of-view’ on eHealth already in 2015 [2]. Likewise, WONCA published a ‘Policy Statement on eHealth’ in 2016 [3]. Several ‘quick and dirty’ searches using PubMed, revealed over a hundred international articles on eHealth (mHealth, telemedicine, electronic medical records, digital health, etc.) in primary care, on an overwhelming variety of topics.

Conceptual clarity was provided by a (Dutch) ‘whitepaper’ on the concepts of eHealth [4]. It introduced three dimensions to classify eHealth technology: setting (public health, clinical healthcare, and healthcare support), users (citizen/patient, healthcare professional, and others) and technology (e.g. websites, portals, apps, self-measurement devices, domotics (home automation), electronic health record systems, secure gateways, etc.) [4]. I learned that, although the focus of eHealth is on the patient interacting with the healthcare system, there is international consensus on a definition of eHealth that covers a much broader spectrum of activities: ‘eHealth is the use of (digital) information and communication technology (ICT), in particular, internet technology, to support or improve health and healthcare’ [4–6].

Following that definition, I guess that many family physicians who do not consider themselves as ‘early adopters’ of eHealth are using (doing?) it already. You probably have an electronic medical information system, in which you record your consultations, your test results, your diagnoses, your medication; and the transactions you can declare. Your system may communicate with the local pharmacy. Perhaps you can request laboratory tests directly from your information system, and send electronic referral letters to specialists using relevant information from the patient’s medical record. You also may receive the test results from the laboratory and letters from specialists digitally, through a secure internet connection, directly into your information system. Furthermore, many of us are getting used to patients who have prepared themselves for the consultation using health information from the internet, or who show you a picture on their smartphone of how a wound, swelling or skin rash looked a few days ago. Some patients may visit you with the results of their home blood pressure measurements or glucose level readings, either on a paper note or maybe as an excel file on a USB-stick. And you now and then will consult a clinical guideline on the website of your society, or fill in a diagnostic decision rule – such as the Wells rule for pulmonary embolism or the Cha_2_dsvas_2_c score – using an app on your smartphone. You may explain something to a patient showing him/her a website with valid information, an illustrative graphic, or an instructive video clip. In short, chances are high you do a lot more on eHealth than you thought at first sight.

Looking at these examples, it is striking that digital inter-professional communication technology seems more established than digital technology for mutual communication between family physician and patient. Doctors will have few objections to the making of appointments electronically by their patients, but many doctors may not feel at ease treating patients without having seen them. We like to stay on the safe side, even if that is causing loaded consultations hours and long working days. We prefer a live consultation to a telephone conversation. The better we know a patient, and the more stable his/her condition, the more we dare to rely on indirect information to make a medical decision: agree with a request for a repeat prescription, answer an email question (‘e-consultation’) or trust self-measured blood pressure or glucose readings.

When the digital technologies of patient and doctor start communicating with each other, and the doctor or patient will make choices based on that communication, not only strict safeguards for cybersecurity and privacy are required; also high demands are placed on data reliability (self-measurements, online questionnaires). Clear rules on mutual responsibilities – rights and obligations, do’s and do not’s – will have to be developed. Medico-legal issues will need elaboration.

The promise of eHealth is comprehensive: better quality of care, more effective care, more efficient care, better service to the patient, more control for the patient (shared decision making, self-management support) and growing availability of high-quality data for quality assurance, education and research [3]. However, much is still unclear, implying that we need to experiment. We will have to learn from each other's experiences and the experiences of our patients. What digital healthcare can replace part of the healthcare as we now deliver; and what digital healthcare may be an addition to our current healthcare services? And what are the financial consequences of integrating eHealth in everyday healthcare? Health literacy is another perspective that needs attention.

More fundamental questions arise as well. How does eHealth influence the doctor–patient relationship? How does the personal expertise of healthcare professionals relate to algorithm-guided decision making? If hospital specialists efficiently communicate with their chronic patients through a hospital-based patient portal (see the examples above), how does that affect the role of family physicians? Is it okay if we are being bypassed? Should patients have ‘open access’ to their medical records? What are the risks of creating an ever-growing digital healthcare network connecting everyone (‘big data’)?

eHealth is here to stay. How do we, family physicians, engage in this development? Where are the opportunities for us, what are our needs, what are our priorities? What can be evaluated and implemented quickly (e.g. online appointments, online prescription requests, and e-consultation), what topics require more extensive experiments (e.g. digital support of self-management by patients, granting patients digital access to their medical record) [2]? The recently published ‘National General Practice Research Agenda’ included a table (see Box 1) on the topic ‘eHealth’, which gives an idea of what GPs in the Netherlands consider relevant eHealth topics [7].

Box 1 The top-ten research topics on eHealth in the Dutch National General Practice Research Agenda* [7].

**Table:** 

How can e-health innovations in primary care be evaluated on safety, applicability and cost-effectiveness? What demands should e-health innovations meet?
How can the GP’s information system (and ICT in general practice in general) contribute to better, safer, more efficient and cheaper healthcare? (e.g. through ICT use in triage, expert systems, etc.)
What is the effectiveness of new ways to implement lifestyle interventions?
How can general practitioners optimally apply pharmacogenetics and pharmacogenetic knowledge? What is needed for this?
Are home measurements and teleguidance of heart failure in the home situation feasible and effective in preventing hospital admissions?
In what way can the GP effectively collaborate in networks of healthcare providers?
How can the patients' self-direction be best designed in issues like viewing their medical record, making appointments and shared decision-making?
How effective are e-health applications in general practice, particularly in chronic conditions and mental health disorders?
What is the significance of self-tests (home measurements) for the quality of GP care?
What is the potential role of e-health for the physician-patient relationship?

* translated by JS.

To address the many challenges raised by the phenomenon of eHealth, we need an open mind: what technology is there? What promise does it hold? What impact could it have? How would that change our routines? How do we value that? Are we ready for that? What conditions must be met before we can implement an innovation? We also need a critical attitude: what technology works, in what settings, for what patients, and under what conditions? How can it be made safe?

The *European Journal of General Practice* will be happy to be a scientific vehicle for original studies, reviews and opinion papers on eHealth. In the past, this Journal has published articles on telemedicine and home telemonitoring, respectively [8,9]. To stimulate further publications on eHealth in primary care, we are planning a Series on this topic to be published in the coming two years. So, if you are interested in eHealth – and you should – keep an eye on this Journal.
